# Protocol to analyze the bacterial pangenome using PAN2HGENE software

**DOI:** 10.1016/j.xpro.2022.101327

**Published:** 2022-04-15

**Authors:** Pablo Henrique Caracciolo Gomes de Sá, Jorianne Thyeska Castro Alves, Adonney Allan de Oliveira Veras

**Affiliations:** 1 Federal Rural University of Amazonia Campus Tomé-Açu (UFRA), Pará, Brazil; 2 Pará State University Campus Marabá (UEPA), Pará, Brazil; 3 Faculty of Computing, Federal University of Pará Campus Castanhal (CCAST-UFPA), Pará, Brazil

**Keywords:** Bioinformatics, Sequence analysis, Genetics, Genomics, Microbiology

## Abstract

The PAN2HGENE is a computational tool that enables two main analyses. First, the tool can identify gene products absent from the original prokaryotic genome sequence. Second, it enables automated comparative analysis for both complete and draft genomes. All analyses are performed through a simple and intuitive graphical user interface without the need for extensive and complex command lines.

For complete details on the use and execution of this protocol, please refer to Silva de Oliveira (2021).

## Highlights

• Identify new gene products and perform comparative analysis
• Improve representation of gene products and explore all content present in raw reads
• Facilitate analysis with reports and graphs produced with the R tool

## Graphical abstract

**Figure:**
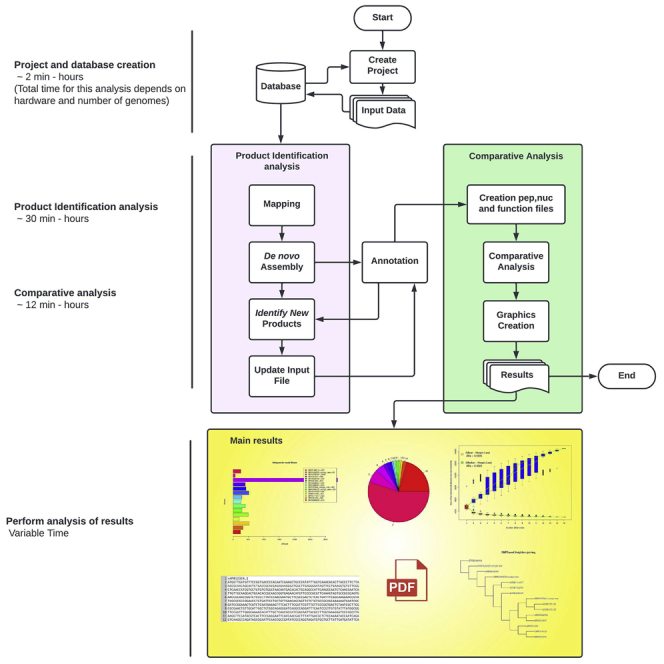

## Before you begin

The PAN2HGENE is a computational tool that performs two main analyses: 1- the identification of new gene products that are not represented in the original genomic sequence. Despite the high accuracy of genome assembly software, none of them is 100% accurate in generating the result. When mapping the raw reads against an assembly result or even the file containing a complete genome, it was observed that several reads do not show any match. With the assembly of these unmapped reads followed by an annotation, it was possible to observe gene products that were absent from the original genomic sequence used as input for analysis. And 2- comparative analysis for both complete and draft genomes. Both analyzes are performed automatically.

The pipeline has three execution modes, complete with all modules or executing each analysis separately (only the identification of new gene products or only the comparative analysis.).

PAN2HGENE is equipped with an intuitive graphical interface that facilitates the execution process without the need to use extensive and complex command lines, the results consist of reports and graphs aimed at helping the user’s analysis process.

## Key resources table

**Table:** 

REAGENT or RESOURCE	SOURCE	IDENTIFIER
**Deposited data**		

Fasta files	https://www.ncbi.nlm.nih.gov	GenBank: AP012324.1, LR134348.1, NZ_JACZEM010000001.1
SRA files	https://www.ebi.ac.uk	EMBL-EBI: SRR1151287, DRR017668, SRR611381

**Software and algorithms**		

PAN2HGENE v2.0	(Silva de Oliveira et al., 2021)	https://sourceforge.net/projects/pan2hgene-software
SPAdes v3.15.4	(Bankevich et al., 2012)	http://cab.spbu.ru/software/spades/
PGAP 1.2.1	(Zhao et al., 2012)	https://sourceforge.net/projects/pgap/files/
Patric v1.034	(Wattam et al., 2014)	http://www.patricbrc.org/
Prokka v1.14.6	(Seemann, 2014)	https://github.com/tseemann/prokka

**Other**		

	User computer: A desktop with a fourth-generation Core i5 processor, 16 GB of RAM, and the Ubuntu 20.04 operating system	n/a

## Materials and equipment

This protocol was created using a desktop with a fourth-generation Core i5 processor, 16 GB of RAM, and the Ubuntu 20.04 operating system. PAN2HGENE has also been successfully tested on Debian and Mint operating systems. So, if the user wanted to reproduce the protocol in the Debian distribution, it will be necessary to log in as root and then remove the sudo command from the beginning of the execution of each protocol command.

## Step-by-step method details

### Step 1: install dependencies

**Timing: 1 h**

The complete installation of PAN2HGENE starts with the installation of the system dependencies and then continues with the installation of other programs that are part of the pipeline.

1. To start the installation run the commands in the Box 1. Below to update the system.

**Box 1**

sudo apt-get install make

sudo apt-get install build-essential

sudo apt-get install curl

**CRITICAL:** If you face any problem with the commands of the Box 1, we suggest update your system with the commands ‘sudo apt-get update’ and ‘sudo apt-get upgrade’. After this try to run the Box 1 again.

2. Then we will install some packages and programs with the commands below.

**Box 2**

sudo cpan install YAML.pm

sudo cpan install YAML::XS

sudo cpan install Bio::AlignIO

sudo cpan install Statistics::LineFit

sudo cpan install Statistics::Distributions

sudo cpan install Bio::Perl

sudo cpan install Bio::SearchIO:::hmmer3

sudo apt-get install screen

sudo apt-get install bowtie2

sudo apt-get install blast2

sudo apt-get install samtools

sudo apt-get install python3-distutils

sudo apt-get install python

sudo cpan install DBI

sudo apt-get install mafft

sudo apt-get install mcl

sudo apt-get install phylip

### Step 2: installation of software that compose the pipeline

**Timing: 2 h 22 min 30 s**

The following are the steps to install the external software that are part of the PAN2HGENE pipeline.

**Figure 1:**
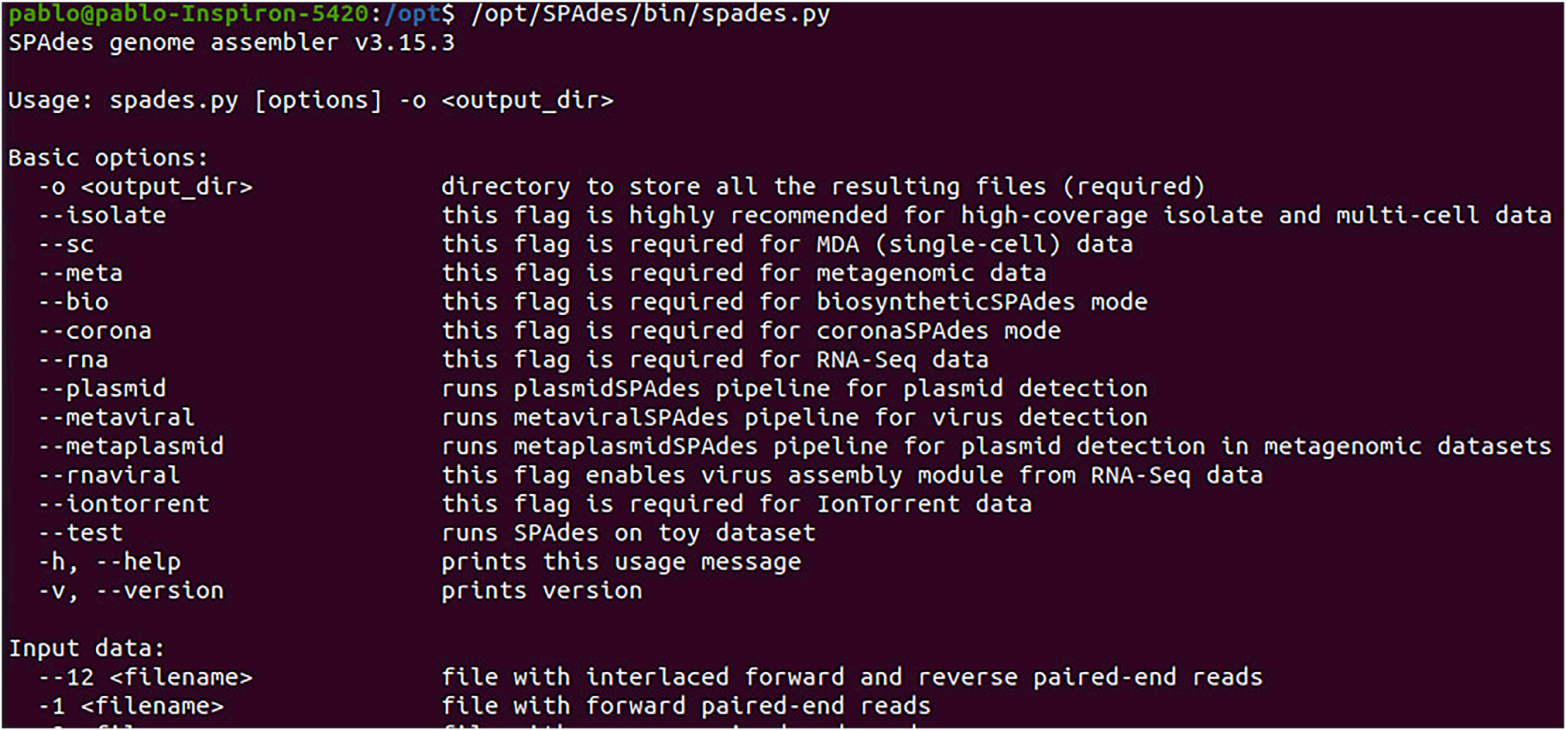
SPAdes test: Demonstrates the SPAdes test run

**Figure 2:**
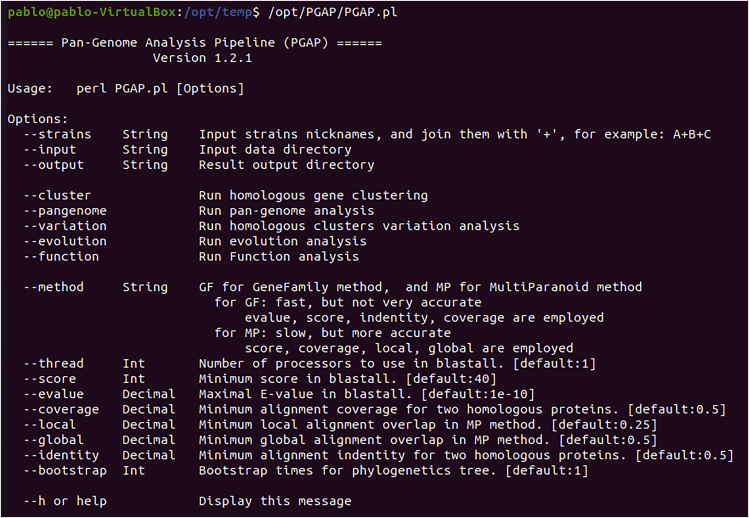
PGAP test: Demonstrates the PGAP test run

**Figure 3:**
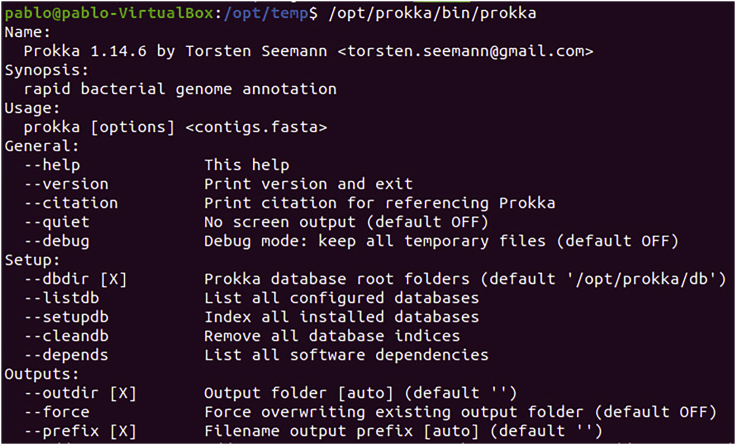
Prokka test: Demonstrates the Prokka test run

**Figure 4:**
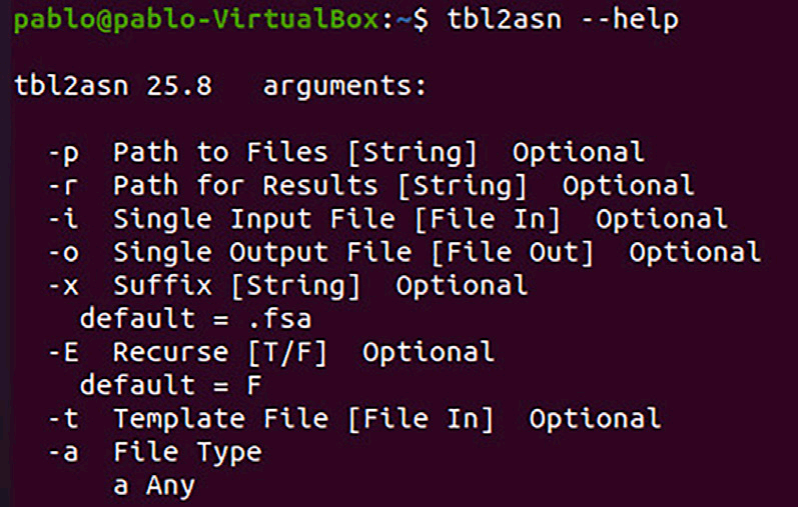
Tbl2asn test: Demonstrates the Tbl2asn test run

**Figure 5:**
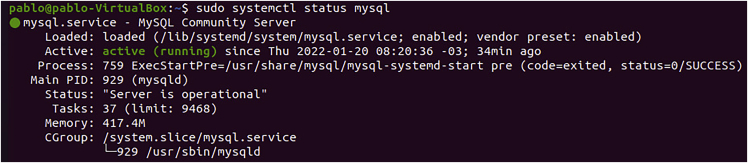
MySQL test: Demonstrates the MySQL test run

3. **SPAdes Installation.** SPAdes is available at http://cab.spbu.ru/software/spades/, the installation process follows below.
  **Box 3**
  wgethttp://cab.spbu.ru/files/release3.15.3/SPAdes-3.15.3-Linux.tar.gz
  tar -xzf SPAdes-3.15.3-Linux.tar.gz
  a. At the end of the installation, the user must move the SPAdes folder to the /opt directory.
    **Box 4**
    mv SPAdes-3.15.3-Linux/ SPAdes/
    sudo mv SPAdes/ /opt/
    cd /opt/
    sudo chmod 777 -R SPAdes/
  b. To validate the installation, run the command (see Box 5), shown in Figure 1.
    **Box 5**
    /opt/SPAdes/bin/spades.py

4. **PGAP Installation.** The PGAP software is available at https://sourceforge.net/projects/pgap/, after downloading it follow the steps below, it is important to note that at the time of building this protocol the most current version was PGAP-1.2.1.
  **Box 6**
  tar -xzf PGAP-1.2.1.tar.gz
  mv PGAP-1.2.1/ PGAP/
  sudo mv PGAP/ /opt/
  cd /opt/
  sudo chmod 777 -R PGAP/
  a. The following step is the configuration of the PGAP script (/opt/PGAP/PGAP.pl), it can be done using gedit or other text editor preferred by the user.
    **Box 7**
    sudo gedit /opt/PGAP/PGAP.pl
  b. Modify the path of the software leaving the lines of the file the same as the lines without comments (lines that do not start with the # character). It is necessary to adjust the execution path of the programs e.g., /usr/bin/formatdb according to the path where the software is in your operating system. In Box 8 below, the lines that do not start with the character # demonstrate the configuration performed in the Ubuntu operating system.
    **Box 8**
    ### programs from BLAST
    my $formatdb="/usr/bin/formatdb";
    my $blastall="/usr/bin/blastall";
    ### programs from mcl
    my $mcl="/usr/bin/mcl";
    ### programs from mafft
    my $mafft="/usr/bin/mafft";
    ### programs from PHYLIP
    my $seqboot="/usr/lib/phylip/bin/seqboot";
    my $neighbor="/usr/lib/phylip/bin/neighbor";
    my $consense="/usr/lib/phylip/bin/consense";
    my $dnaml="/usr/lib/phylip/bin/dnaml";
    my $dnadist="/usr/lib/phylip/bin/dnadist";
    my $dnapars="/usr/lib/phylip/bin/dnapars";
  c. Look for the 3 lines below and replace "./" with "/opt/PGAP/". After the replacement the lines should look like Box 10.
    **Box 9**
    system("perl ./multiparanoid.pl -species ".join(".pep+",@species).".pep -unique 1");
    system("perl ./Blast_Filter.pl All.blastp All.pep $coverage $identity $score | $mcl - --abc -I 2.0 -o All.cluster");
    system("perl ./inparanoid.pl $blastall $thread $formatdb $score $global $local
    $species[$i].pep $species[$j].pep");
    **Box 10**
    system("perl /opt/PGAP/multiparanoid.pl -species ".join(".pep+",@species).".pep -unique 1");
    system("perl /opt/PGAP/Blast_Filter.pl All.blastp All.pep $coverage $identity $score | $mcl - --abc -I 2.0 -o All.cluster");
    system("perl /opt/PGAP/inparanoid.pl $blastall $thread $formatdb $score $global $local $species[$i].pep $species[$j].pep");
  d. Save the file and close gedit. To validate that PGAP was installed correctly, run the command below and result should be similar to this (Figure 2).
    **Box 11**
    /opt/PGAP/PGAP.pl

5. **Prokka Installation.** The Prokka software is available https://github.com/tseemann/prokka, to install the version for Ubuntu/Debian/Mint run the commands below.
  **Box 12**
  sudo apt-get install libdatetime-perl libxml-simple-perl libdigest-md5-perl git default-jre bioperl
  sudo cpan Bio::Perl
  sudo git clonehttps://github.com/tseemann/prokka.git/opt/prokka
  sudo chmod 777 -R /opt/prokka
  /opt/prokka/bin/prokka --setupdb
  sudo chmod 777 -R /opt/prokka
  a. To verify that Prokka was installed correctly, run the command below and the result should be similar to this (Figure 3).
    **Box 13**
    /opt/prokka/bin/prokka

6. **R Installation.** The R software is installed by following the commands below.
  **Box 14**
  sudo apt-get update
  sudo apt-get install r-base r-base-dev
  a. After installation, run R and install the libraries.
    **Box 15**
    sudo R
    install.packages('ape')
    install.packages("plotrix")
    install.packages("minpack.lm")
    install.packages('ctv')
    library('ctv')
    install.views('Phylogenetics')
    update.views('Phylogenetics')
    q()

7. **Tbl2asn Installation.** The tbl2asn tool is installed by running the following commands.
  **Box 16**
  wget -N
  ftp://ftp.ncbi.nih.gov/toolbox/ncbi_tools/converters/by_program/tbl2asn/linux64.tbl2asn.gz
  gunzip linux64.tbl2asn.gz
  sudo chmod +x linux64.tbl2asn
  mv linux64.tbl2asn /usr/local/bin/tbl2asn
  a. And to test if everything is correct, run the command below and the result should be similar to this (Figure 4).
    **Box 17**
    tbl2asn --help

8. **MySQL Installation.** To install MySQL server run the following commands.
  **Box 18**
  sudo apt-get install mysql-server
  sudo mysql_secure_installation
  a. MySQL will ask you to create a password for the root user. Enter the password and answer Y when asked.
  b. The component checks to see if the new password is strong enough. Choose one of the three levels of password validation:
    i. Low. A password containing at least 8 characters.
    ii. Medium. A password containing at least 8 characters, including numeric, mixed case characters, and special characters.
    iii. Strong. A password containing at least 8 characters, including numeric, mixed case characters, and special characters, and compares the password to a dictionary file. Enter 0, 1, or 2 depending on the password strength you want to set.
  c. The script then prompts for the following security features: Remove anonymous users? Disallow root login remotely? Remove the test database and access to it? Reload privilege tables now?
  d. To check if your MySQL was installed correctly run the command below and the result should be similar to this (Figure 5).
    **Box 19**
    sudo systemctl status mysql

### Step 3: using PAN2HGENE

**Timing: 1 h 27 min**

After installing all dependencies, the user must download PAN2HGENE. To start using it, follow the steps below.

9. **PAN2HGENE Download.** The PAN2HGENE jar package is available at (https://sourceforge.net/projects/pan2hgene-software/). Download the pan2hgenev2.0.jar and lib_v2.tar.xz files leaving both in the same directory. See the example below. The PAN2HGENE pipeline can be executed in three different ways, each one performing a specific analysis.

**Box 20**

cd /home/pablo/panTest/

tar -xf lib_v.2.tar.xz

sudo chmod 777 -R /home/pablo/panTest/

java -jar pan2hgenev2.0.jar

**Figure 6:**
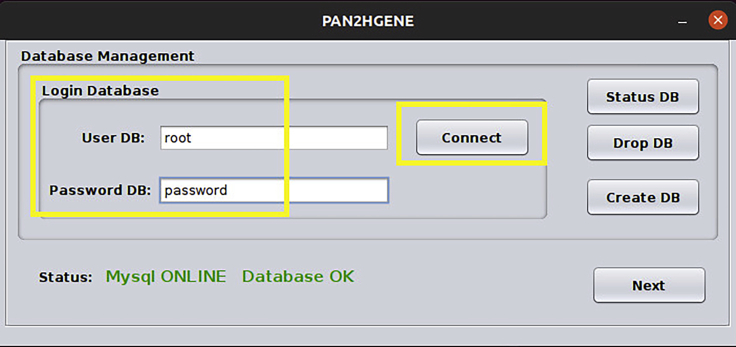
Database management: The window has all functions to handle database, like status, drop, create e connect database
  b. On the following screen, it is necessary to enter the project name and select the type of analysis to be performed. In the following example, the name “Test1” and the Product Identification option were added, after that press the New button (Figure 7).

10. **PAN2HGENE Product Identification analysis.** The input for this analysis is a pair of files in FASTA and FASTQ format, the FASTA file can contain a complete genome or a draft of the organism. An attempt is made to identify possible new gene products for the analyzed genomes.
  ***Note:*** In this example, product identification analysis is performed using a *Bifidobacterium breve* DSM20213 genome and paired reads from the Illumina HiSeq 2000. To start the Product Identification analysis, place the fasta genome and fastq reads in the same folder, follow the steps below.
  **Box 21**
  java -jar pan2hgenev2.0.jar
  a. If this is your first use, enter the root user and password and press the create DB button, else enter the root user and root password in the indicated fields and then click the Connect button, then click on the Next button (Figure 6).

**Figure 7:**
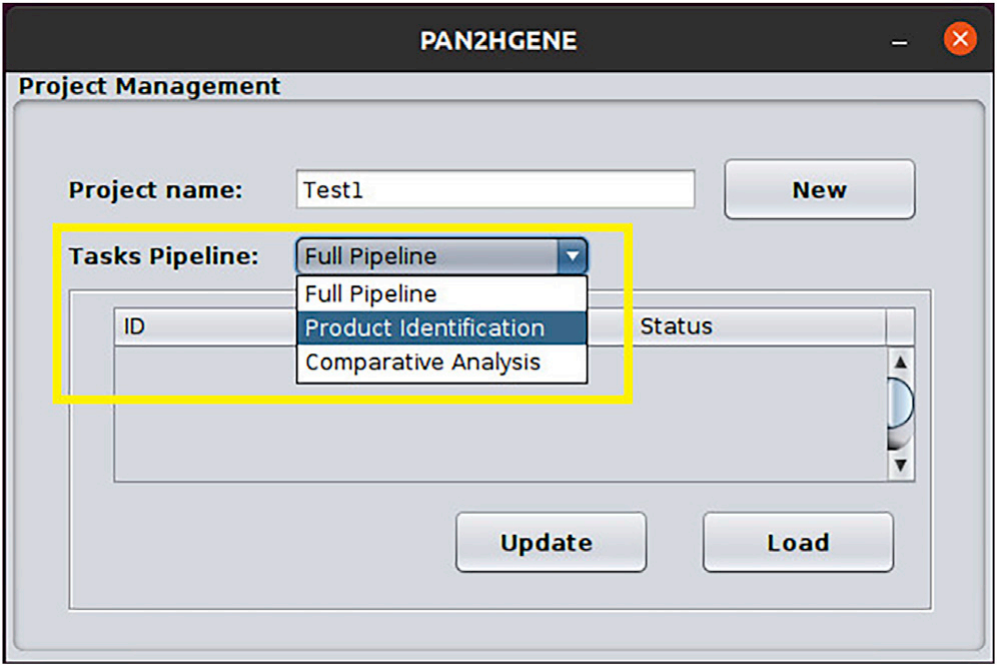
Project management: In this window, the user will create the project, so just inform the name of the project and the type of analysis to be performed
  c. Data input is done in the following window. Press the Browse button to select the FASTA file (Remember that fasta genome and fastq reads must be in the same folder). The reads files will be displayed below, select the appropriate reads for the organism, inform the type of reads and if it is paired, inform the order and orientation (Figure 8).

**Figure 8:**
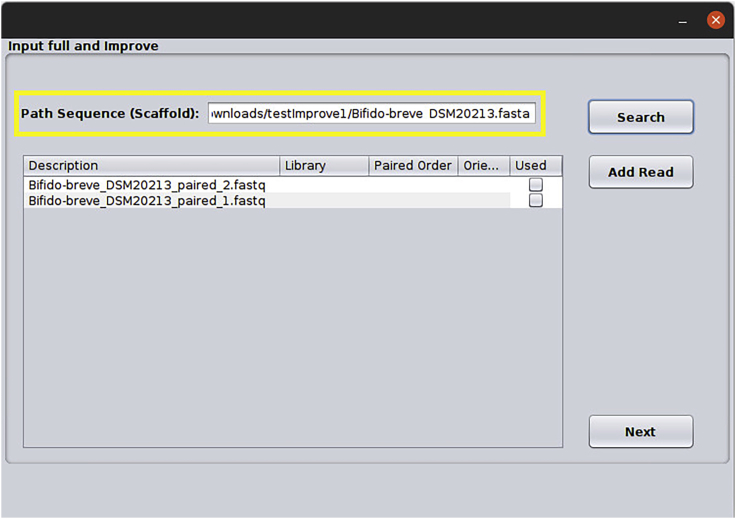
Input data: In this window, the user will add the files in fasta and fastq format depending on the analysis chosen
  d. Press Add Read button and confirmation message will be displayed (Figure 9).

**Figure 9:**
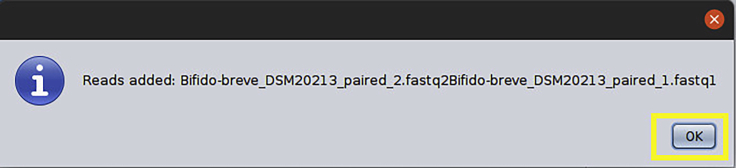
Confirmation warning
  e. Note that the reads are now marked as used, repeat the same process if there are more genomes. Then click Next (Figure 10).

**Figure 10:**
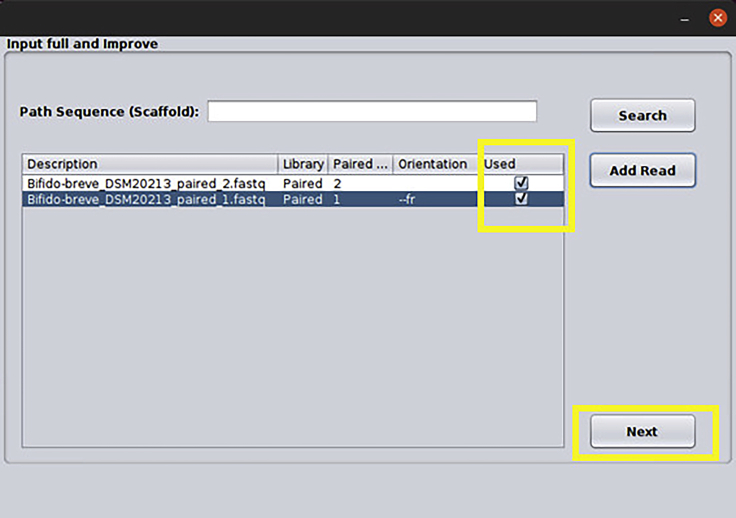
Input data confirmation: Demo files after being added to the analysis
  f. The screen below will be shown, here it is possible to modify the parameters of PAN2HGENE for Bowtie, Comparative Analysis, and Annotation process. In this case, we will use the default parameters. So just click the Save Data button, then click Next (Figure 11).

**Figure 11:**
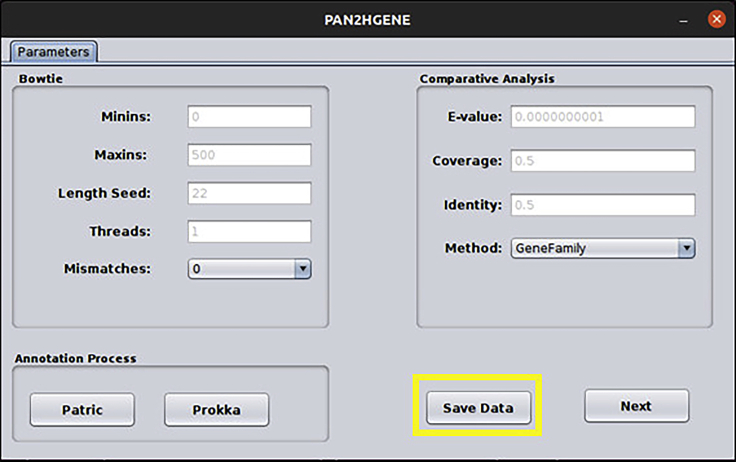
Parameters window: In this window, the user can edit the parameter values or can choose to use the default value
  g. The screen below will be shown. To start the analysis, click on the Perform analysis button. And in the Logs field, it is possible to check the analysis steps being performed. When the analysis is finished, the message Complete Analysis will appear in the Logs field, as can be seen on the screen below. Now close PAN2HGENE and go to the folder where the data was analyzed (Figure 12).

**Figure 12:**
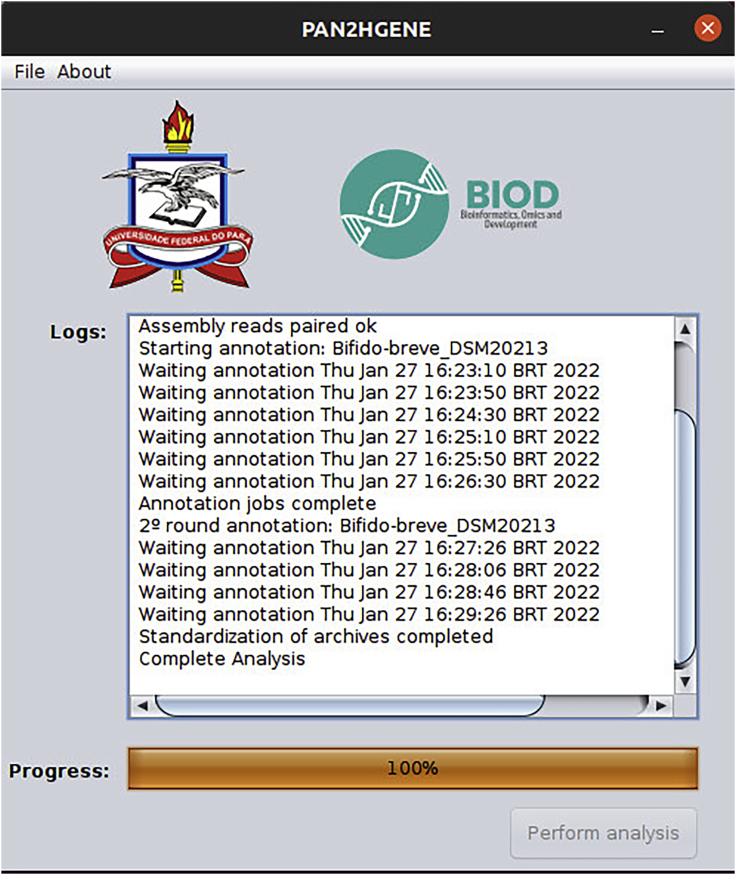
Perform analysis and Log: In this window, the user executes the analysis and follows the execution steps
  h. Several files will be inside the folder, in addition to the fasta genome and the fastq reads used in the analysis. The result of the Product Identification analysis are the three files marked below, GenomeNameBlastResult_Products.fasta, GenomeNameBlastResult_report.pdf, and GenomeNameBlastResult_Report.txt (Figure 13).

**Figure 13:**
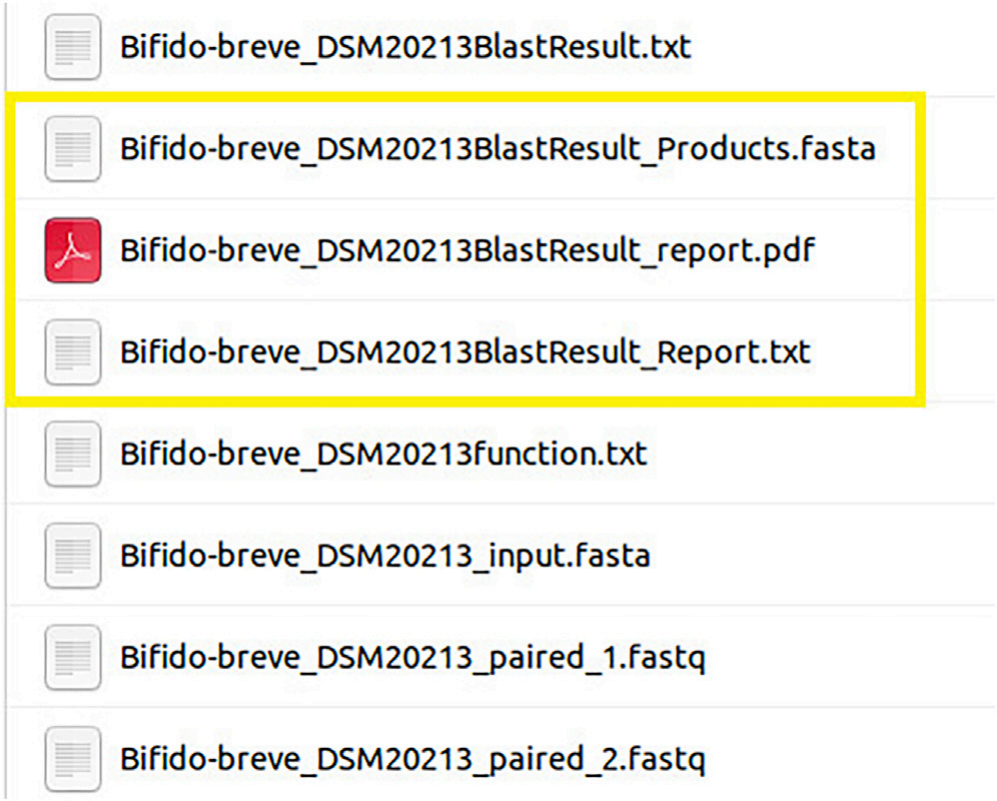
Product identification analysis results

**Figure 14:**
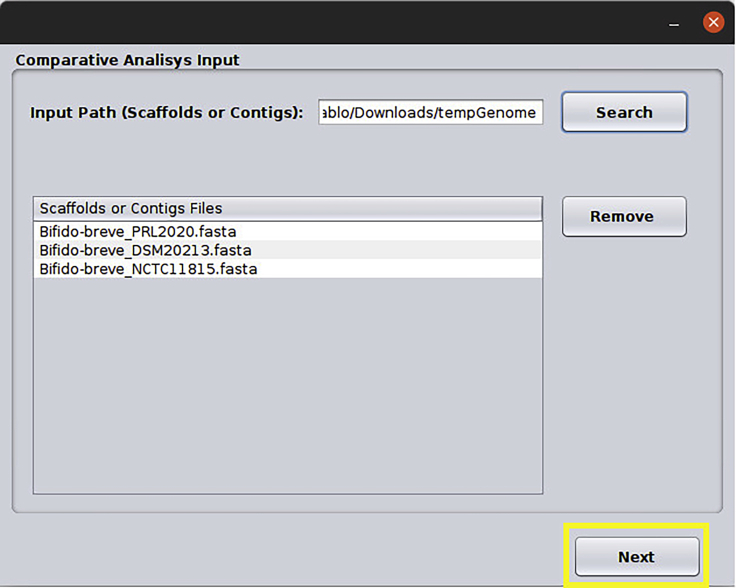
Input data window: In this window, the user input complete or draft genomes in FASTA format
  b. The analysis results are organized in the pgfiles directory. The files that are the results of the Comparative Analysis are the files that start with the numbers 1, 2, 3, 4, 5 and the figures in PNG format (Figure 15).

11. **PAN2HGENE Comparative analysis.** In this example, three fasta genomes, *Bifidobacterium breve* DSM20213 (complete), *Bifidobacterium breve* NCTC11815 (complete) and *Bifidobacterium breve* PRL2020 (draft with six contigs) were used. To start Comparative Analysis, place all fasta genomes in the same folder as shown below.
  **CRITICAL:** The initial steps are the same as shown in Figure 6, Figure 7, Figure 8, Figure 9, Figure 10, Figure 11, Figure 12, with the exception of the input data window that changes for this specific analysis.
  a. When informing the directory of the FASTA files, they will be listed as shown in the figure below (Figure 14).

**Figure 15:**
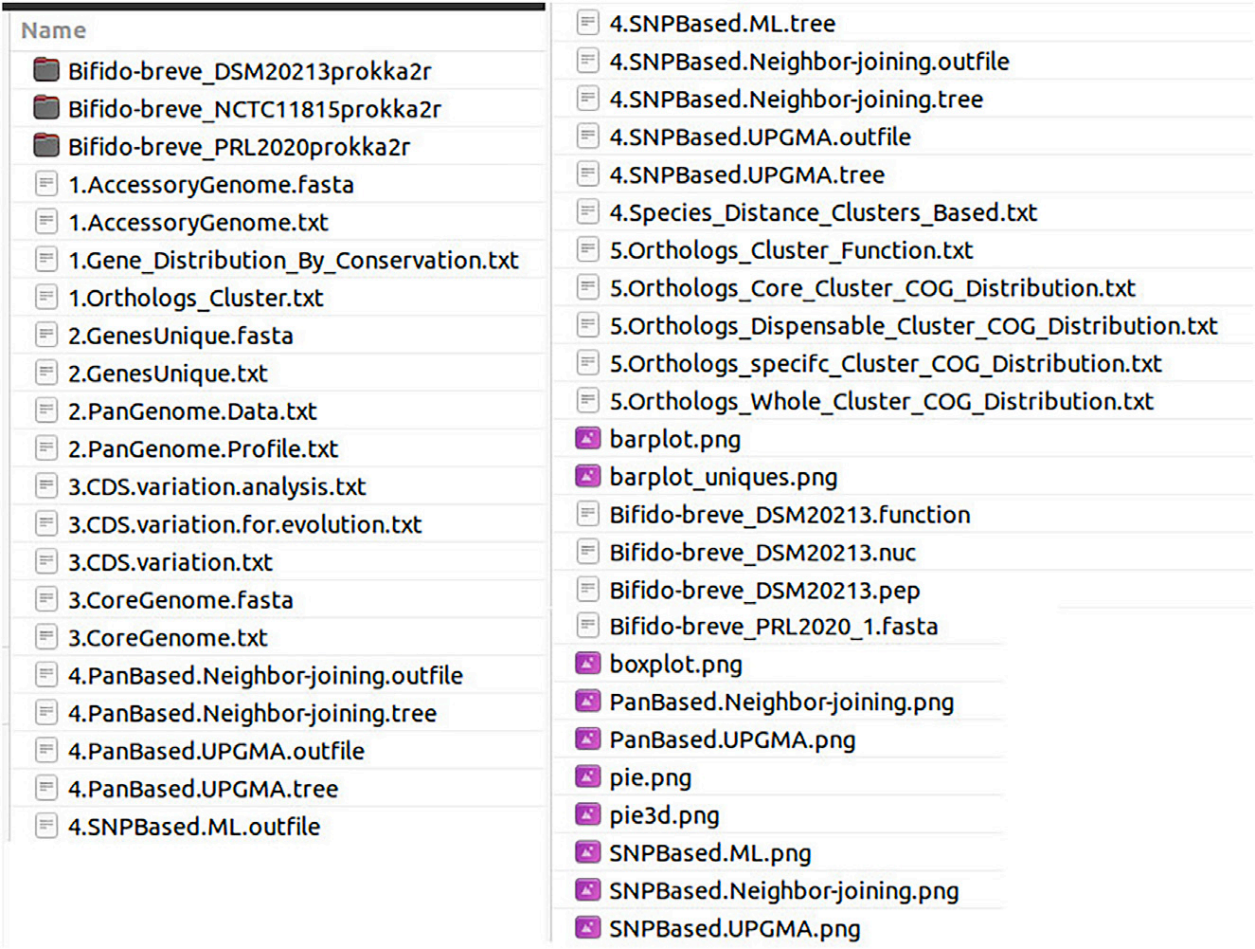
Comparative analysis results

12. **PAN2HGENE Full pipeline.** The Full Pipeline analysis performs Product Identification analysis and Comparative Analysis automatically and sequentially. Thus, the new gene products identified in the Product Identification step will be used in the Comparative Analysis step.

**Figure 16:**
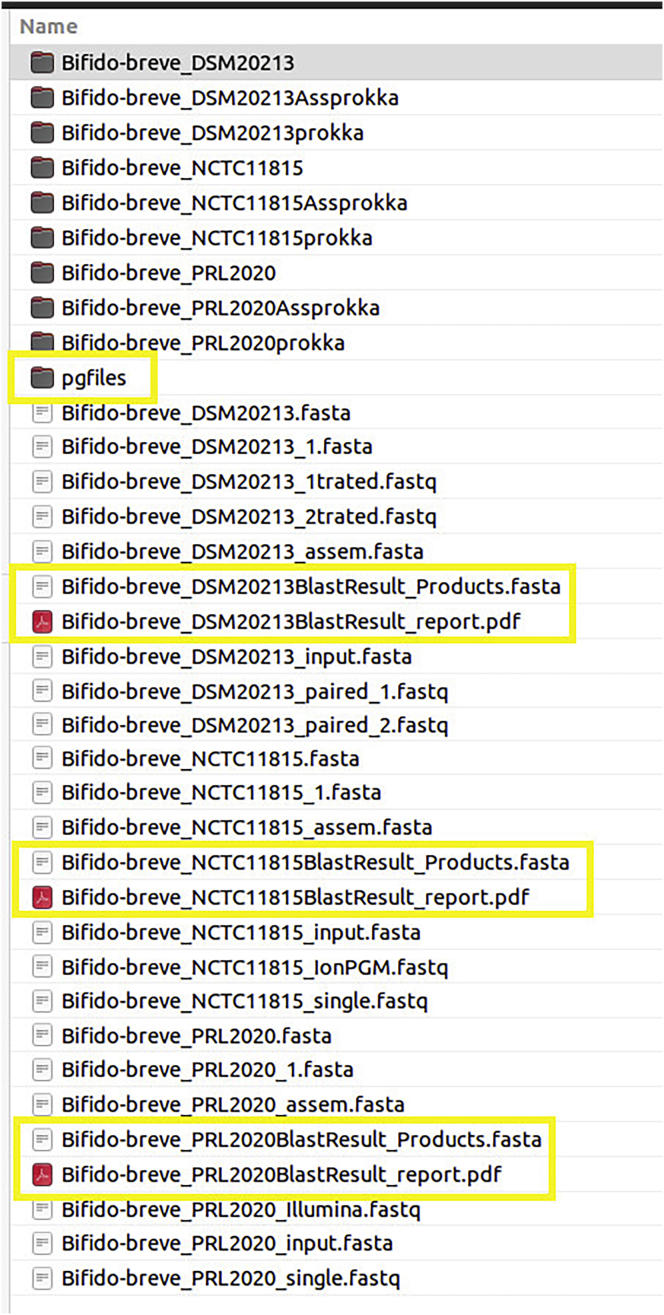
Full pipeline analysis results

***Note:*** Now follow the steps described previously in the item ‘10. PAN2HGENE Product Identification analysis’, selecting Full Pipeline analysis instead of Product Identification (Figure 16).

**Figure 17:**
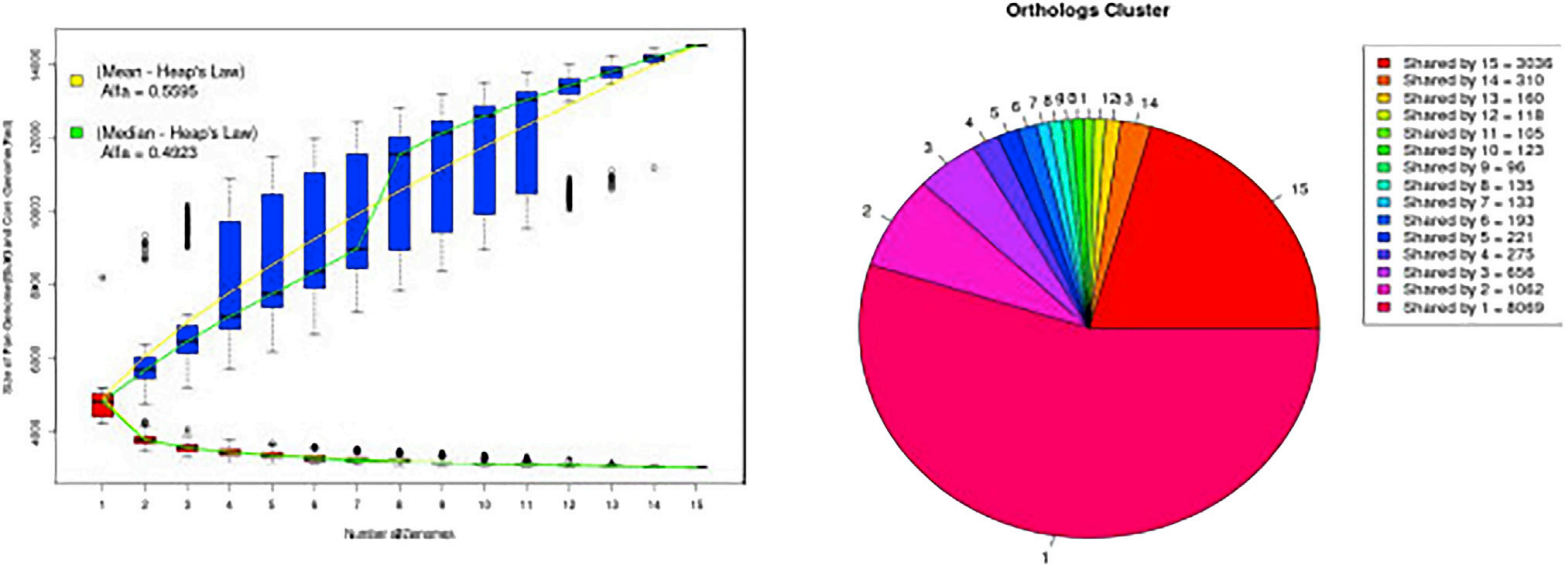
Graphics results of pangenome: On the left we have the graph with the information about pangenome, together with the Heap’s Law calculation and on the right, in the pie chart, the information about the amount of unique gene products and genes shared between the analyzed organisms

**Figure 18:**
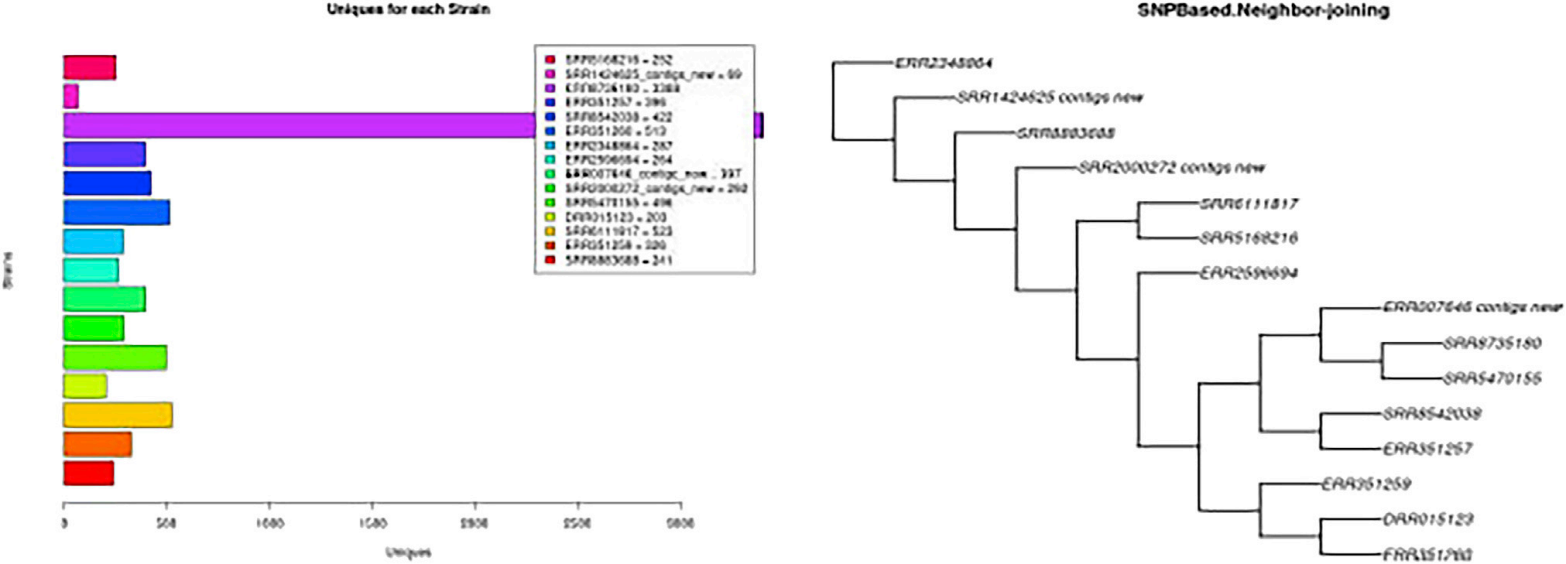
Graphics results of unique genes: On the left we have the graph with information about the amount of unique genes per organism present in the analysis and on the right an example of a phylogenetic tree graph

13. **Main graphical results.** The graphs are produced by running the comparative analysis, so the creation is included in the processing time (Figures 17 and 18).

### Step 4: patric optional usage

**Timing: 2 min**

Patric software has been integrated into the pipeline as an alternative to automatic annotation software. Thus, the user is free to choose between Patric or Prokka, for the annotation execution.

**Figure 19:**
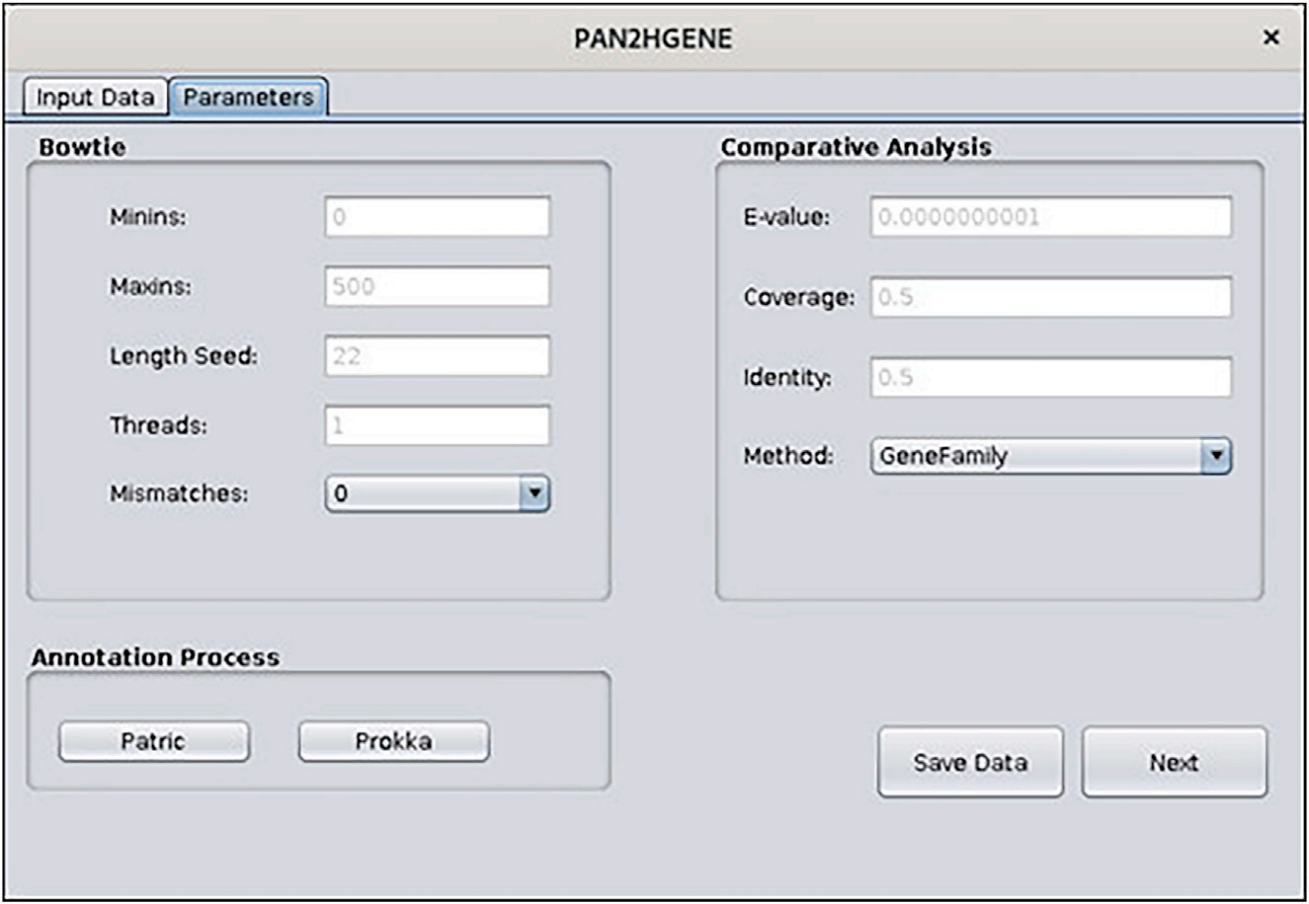
Input data parameters: The user can select Patric annotation tool

**Figure 20:**
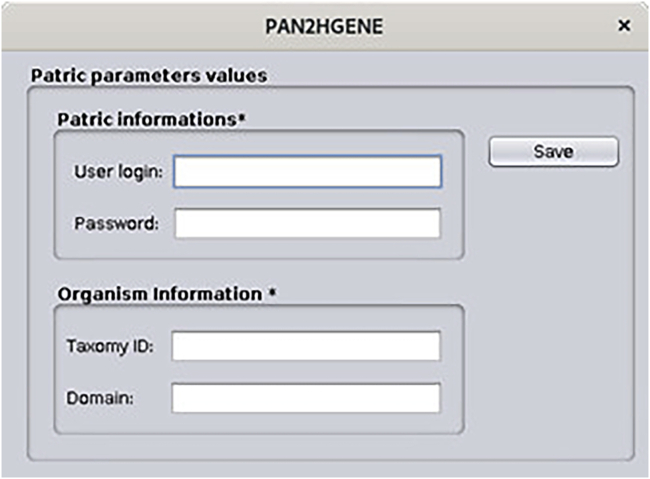
Parameter patric: The user enter with your credentials, taxonomy ID and Domain organism, for instance, Escherichia coli, Taxonomy ID 562 and Domain Bacteria

14. Your PAN2HGENE is now ready to use, If the user does not want to use Patric to make the annotation, it is not necessary to perform the following steps.
  ***Note:*** However, the PAN2HGENE offers the option to perform all annotation analyses through PATRIC instead of Prokka (which is the default option). If you want to use PRATIC in the annotation process, follow the steps below. If you do not already have a PATRIC account, you will have to register on (https://patricbrc.org/).
  a. Install PATRIC Command Line Interface.
    **Box 22**
    curl -O -Lhttps://github.com/PATRIC3/PATRIC-distribution/releases/download/1.034/patric-cli-1.034.deb
    sudo dpkg -i patric-cli-1.034.deb
    sudo apt-get -f install
  b. If you prefer you can also install PATRIC using the tool gdebi.
    **Box 23**
    sudo apt-get install gdebi-core
    sudo gdebi patric-cli-1.034.deb
  c. Setting, copy the file “p3-login.pl”, provided with the pan2hgene files, and replace it in the installation directory of Patric-cli.
    **Box 24**
    cp -r < file directory > usr/share/patric-cli/deployment/plbin/
  d. When performing any of the PAN2HGENE analyses, it is possible to choose the Patric annotation instead of the Prokka annotation. Before saving the parameters, click on the PATRIC button (Figures 19 and 20).

## Expected outcomes

Although there are several tools to perform the comparative analysis, PAN2HGENE stands out for its characteristics, presenting a simple graphical interface to facilitate the analysis, instead of complex command lines. This tool can perform the identification of possible new gene products in a genome and can also perform, unlike other tools, the comparative analysis using complete genomes and draft genomes. The results are presented graphically and/or textually, without the need for the user to use other programs to analyze or interpret the results.

Finally, it is important to note that both analyzes are performed automatically. And that the input data are fasta genomes and their reads in fastq, without the need for the user to create standardized inputs or need to manipulate input files (PAN2HGENE already does this automatically too).

## Limitations

PAN2HGENE is able to perform comparative analysis using complete genomes and draft genomes as input. The PAN2HGENE will perform an automatic annotation through the Prokka or Patric. At this point, it is important to emphasize that the annotation does not depend on the PAN2HGENE, as the pipeline only uses the annotation generated by Prokka or Patric.

## Troubleshooting

### Problem 1

The figures generated as a result do not present information on the pangenome distribution.

### Potential solution

This usually happens when few genomes are used in comparative analysis. PAN2HGENE can perform comparative analysis for a minimum of 3 genomes. In general, when 3 or 4 genomes are used in the analysis, the pangenome result has practically no distribution information. Thus, the solution is to add a greater amount of genomes in the analysis, remembering that PAN2HGENE does not have a maximum limit of genomes that it can analyze.

### Problem 2

PAN2HGENE never gets to the end of the analysis, it just keeps processing.

### Potential solution

Comparative analysis is a type of exponential analysis, in which the more genomes are inserted into the analysis, the greater the computational cost to perform it. What could be happening is that the computer used is not able to process the analysis. So, the solution, in this case, would be to perform an analysis with fewer genomes, to ensure that PAN2HGENE is working correctly and then try to use a more powerful computer to perform the analysis with all the selected genomes. For example, using a desktop with a fourth-generation Core i5 processor and 16 GB of RAM, when performing the comparative analysis for 10 genomes, the complete run took approximately one and a half hours. And using the same computer to analyze 20 genomes, the complete runtime was approximately 4 h and 20 min.

***Note:*** Problems 1 and 2 are related to the amount of genomes used and the hardware capacity used, respectively, and not to the PAN2HGENE software.

## Resource availability

### Lead contact

Further information and requests should be directed to the lead contact, Allan Veras (allanveras@ufpa.br).

### Materials availability

Results of the application describing the protocol are reported in Silva de Oliveira et al., 2021.

## Data Availability

The published article includes code generated and all used datasets are available at NCBI and described in the key resources table.
